# Reinforcing Science and Policy, With Suggestions for Future Research: Comment on "Evidence-Informed Deliberative Processes for Health Benefit Package Design – Part II: A Practical Guide"

**DOI:** 10.34172/ijhpm.2022.7398

**Published:** 2022-07-13

**Authors:** Anthony J. Culyer

**Affiliations:** Department of Economics and Related Studies and Centre for Health Economics, University of York, York, UK.

**Keywords:** HTA, Deliberation, Consensus, Political Bias

## Abstract

Oortwijn et al continue their guide to good practice in the use of deliberative processes in health technology assessment (HTA) based on a survey of international practice. This is useful, and I applaud their care in maintaining objectivity, especially regarding the treatment of moral and politically controversial issues, in reporting how jurisdictions have handled such matters in designing HTA procedures and in their execution. To their suggestions for future research, I add: the historical development of deliberation in healthcare decision-making and in other fields of public choice, with comparisons of methods, successes and failures; development of guidance on the design and use of deliberative processes that enhance decision-making when there is no consensus amongst the decision-makers; ways of identifying and managing context-free and context-sensitive evidence; and a review of high-level capacity building to raise awareness of HTA and the use of knowledge translation and exchange (KTE) and deliberation amongst policy makers, especially in low and middle-income countries.

 Oortwijn, Jansen and Baltussen (henceforth OJB) continue their practical guide on promoting the use of deliberative processes in health technology assessment (HTA),^1^ initiated in 2021,^2,3^ by making recommendations for good practice based upon a survey of international practice. There is much to commend in these papers, and I have nothing to criticise in either their rapportage or their recommendations. I also applaud the careful way in which they have maintained objectivity, especially regarding the treatment of moral and politically controversial issues, in reporting how various jurisdictions have handled such matters both in the design of HTA procedures and in their execution. This distance is not seen everywhere and, indeed, the philosophical origins of deliberation in public decision-making historically build on strong normative propositions from which it is not easy to escape, and rely also on conceptual precision which is not always in evidence.^4,5^

## Deliberation Is Not the Child of HTA

 It may be true that “increasingly, decision-makers are urged to organise fair, legitimate processes in health benefit package design, with legitimacy referring to the reasonableness of decisions as perceived by stakeholders,” but the urging in many jurisdictions does not come from politically influential circles (academics in particular never fail to urge), and OJB’s assertion that “evidence-informed deliberative processes were developed in response” betrays unfamiliarity with a copious philosophical literature and much practical experience with deliberative processes (evidence-based or other-based) in citizens’ juries, panels and the like.^6^ Nor is it entirely true to say that the design and implementation of deliberative processes is in its infancy.^7,8^

 The explicit use of HTA in the design of health benefits in public insurance plans is, however, a recent phenomenon. Deliberative processes had been discussed before HTA or like methods were formalised and in contexts distant from healthcare and insurance package design, mainly concerning the meanings to be attached to central ideas like “evidence.” Major philosophical contributions were by Habermas,^9^ whose idea of rational discourse as a foundation of liberal democracy has influenced much modern analysis,^10^ Lindblom^11^ on muddling through, and less abstract specific work in non-health territories, some as distant from health policy as waste management.^12^ These earlier developments and applications were highly normative, and continue to have an influence that is not entirely benign.

## Evidence and Science

 HTA operates everywhere in multi-cultures, where absence of mutual understanding and cross-communication can be huge barriers. Consider colloquial and scientific evidence.^12^ Outside the research community, colloquial evidence is anything factual that provides a reason for believing in something. In research, evidence is gathered in order to test hypotheses; evidence is empirical information that is explicit (codified and propositional), systematic (using transparent and explicit methods for codifying), and replicable (so that using the same methods with the same samples generates the same results). Health policy-makers are more likely to use the broadly inclusive, colloquial, definition of evidence, though the evidence-based decision-making movement has engendered a greater (though not always well-understood) regard for scientific forms.^13,14^ Either way, there is a need for decision-makers to possess the competence to interrogate evidence generators, like researchers; there is a need for the evidence itself to be evaluated and interpreted; and there is a need for decisions about who should do the interrogation, evaluation and interpretation. The culture gap can be huge, as can the difference in personal and institutional interests of professional advocates for HTA and deliberation, on the one hand, and of those with decision-making authority on the other. If the latter are to “follow” science, they need first to understand it well enough to be able to interrogate the scientists.

## Context-Free and Context-Sensitive Evidence

 There are two distinct views on the role of science in health system guidance. One (identified closely with evidence-based medicine) is that lab-based science and randomized controlled trials can reveal universal truths. This view provides the basis for context-free guidance. Context-sensitive guidance, on the other hand, is built on the view that evidence has little meaning or importance for decision-making unless it is adapted to the circumstances of its application.^15-17^ This requires evidence on what actually works in the “real world,” when the controls governing scientific experimental methods are relaxed, and when such evidence is combined with evidence from the context of application. Context-free guidance indicates what we understand to work in general. Context-sensitive guidance shows both what works and how (or whether) it might be usefully implemented in the specific circumstances under consideration. In either case, there is a need for processes that enable different types of scientist (clinical, biological, statistical, economic, etc) to work together and, when working in specific circumstances of application, also to work with patients, carers, manufacturers, advisers, managers and decision-makers (ie, those whom we commonly call “stakeholders”) to address issues of implementation, organization, attitudes (of patients, the public, professionals, politicians, or other stakeholders), budgeting, modelling, forecasting, economics, ethics and others.

 Evidence is inherently uncertain, dynamic, complex, contestable, and rarely complete. A process is required to assess its relative merits and limitations in the light of the issue at hand. The deliberative process must be able to access, combine and interpret the population of evidence of all relevant kinds, qualitative and quantitative, context-sensitive or context-free. It should not seek certainty, nor pretend to a wider audience to have found it.

## Evidence, Deliberation and Values

 Deliberative processes can do more than merely combine evidence of widely different kinds. They can also reveal and combine *values*, including evidence about the values held by relevant interest groups and individuals. These values might commonly include a concern for the fairness of a distribution of benefits and harms and how open to external challenge the process and its outcomes ought to be. Some may also, moreover, relate directly to the way in which decisions might impact on specific individuals, especially persons of political importance. OJB recognise this as a context-*free* generalisation, ie, a set of issues that all HTAs need to address, with their resolution being a matter for decision in the specific local context. Context-free guidance needs to be kept distinct from values that are context-sensitive, for example, the value-judgement that HTA ought always to promote greater equality in life-time health status, or one asserting that HTA ought not concern itself with distributional concerns over life-time health status, or that the damage done by traditional healers’ unhygienic methods is tolerable. Such value-judgements may appropriately apply in jurisdiction A but not in jurisdiction B. OJB wisely construct their Box 1 (58 questions to be addressed) as questions, whose answers will usually be context-sensitive, and therefore not answerable as though the context did not matter.

 The presence or absence of consensus amongst the parties to a decision is as much a* consequence* of a deliberative process as it is an *aim* to be sought. OJB state that “reimbursement decisions are ideally reached by consensus” but they also recognise that consensus cannot always be achieved. They fail to note, however, that deliberation may also reveal the reasons for lack of consensus and identify those interests of the people most involved which may facilitate the identification of a more consensual decision, or at any rate a satisfactory politically managed solution. Deliberation can also be usefully informed by sensitivity analyses designed to explore the consequences of making a variety of assumptions about the character and size of potentially context-sensitive factors. Some guidance as to how best to prevent failures to agree and the address those that nonetheless arise, would be a useful further extension of OJB’s work.

## Using Knowledge Translation and Exchange to Create Capacity

 One of the biggest challenges for HTA lies in the related territories of knowledge translation and exchange (KTE), and in building and maintaining the capacity of healthcare systems and the political structures, such as professional societies and public ministries, that support them. To understand HTA and its uses, to commission the work needed for HTA bodies to do their work, and to embrace HTA evidence and reasoning into top level policy decisions plainly requires deliberation.

 A recurrent theme in much writing about progress with the institutionalisation of HTA is the lack of political understanding, or the lack of technical capacity, or both, at high regional or national levels, which is widely recognised as a major barrier to progress.^18^ It is only recently that scientists have begun to learn how to share their research findings other than by publishing in peer-reviewed journals and presenting at conferences to other scientists. A pioneer in developing technologies for genuine KTE has been Toronto’s Institute for Work & Health (https://www.iwh.on.ca), specifically in the field of workplace health and safety, which is the basis for my own hands-on experience with effective KTE.

 The four main components of the IWH strategy are:

- *Building relationships*: formal networks with policy-makers, professional practitioners and clinicians, professional organizations, etc.
- *Building engagement into research*: involving knowledge users and other stakeholders in specific research projects beginning early in the research process, whereby stakeholders can provide guidance in shaping the research question and give information about the context in which research results are likely to be used. It continues to the end of the research project, when stakeholders help craft research messages in ways that are meaningful to the intended audience.
- *Enhancing capacity*: helping external audiences understand and apply research through Systematic Review Workshops, research presentations at which stakeholders can directly learn from, and ask questions of, scientists.
- *Communicating finding*: creating a communications strategy directed at stakeholders. Communication tools might include a central website, newsletters, monthly e-bulletin, plain-language summaries, videos, coverage in general and trade media, and social media (eg, Twitter, LinkedIn and YouTube).

It would be a valuable extension of OJB’s work, to review this and other working experience of KTE (including a genuine *exchange* rather than a unidirectional flow of information from researchers to users).

## The Risk of Politico-Cultural Bias

 Despite their caution, OJB display a systematic preference for transparency and democratic processes. Others may be less circumspect and see in deliberative HTA a political tool for advancing, for example, participative democracy. This removes characteristics like transparency and democratic participation from the context-sensitive category to the context-free type. In my opinion, this would be a mistake. OJB rightly characterise deliberation and HTA as tools to enable decision-makers make legitimate decisions as perceived by those to whom they are accountable but they do not warn of the historical danger of identifying deliberation with a specific world political view (viz. liberal democratic). Most of the world’s populations served by healthcare do not live in democracies, and many jurisdictions do not share a value that exposes decision-makers to critical examination. The prime purpose of some healthcare systems is to make profits. Others seek to maximise human productivity. These features vary contextually and, if HTA is to be a universally helpful tool, it is wrong to require a specific context to form its only proper application. Many believe, as I and OJB do, that a rational consensus developed through discussion is a useful normative guide in liberal democratic policy making. HTA enhances democracy and democratic processes. The same has been claimed of deliberation since Habermas. There is no reason, however, why deliberation may not enhance decision-making in single party states and theocracies or, indeed, even in charitable or for-profit healthcare insurance and delivery systems owned and operated by clinicians, limited liability companies or even traditional healers. It should be a context-free condition that HTA is always and everywhere a *tool *and, as such, ought to be value-neutral and context-sensitive, not weaponized in a broader ideological struggle.

## Future Work

 OJB suggest two main themes for future work in this field. The first is to document more completely the ways in which legitimacy is addressed and decision criteria are chosen; the other is to assess effective impact of using deliberation to design HTA procedures and health benefit packages. I support both proposals and would add a further five:

- The preparation of an account of the historical development of deliberation in healthcare decision-making and in other fields of public choice, with qualitative and quantitative comparisons of methods, successes and failures.
- Further exploration of how best to identify and then manage context-free and context-sensitive evidence (and the design of frameworks for deliberative thinking about them).
- Development of guidance on the design and use of deliberative processes that enhance decision-making when there is no consensus amongst the decision-makers.
- Effective methods of ensuring, when representation is a desideratum, that the usual diversity of social values, to be found in all populations, is appropriately embodied in deliberative groups.
- A major review of high-level capacity building, especially in low and middle-income countries, covering mechanisms such as KTE, and with assessments of (cost-) effectiveness and markers of likely success.

## Ethical issues

 Not applicable.

## Competing interests

 Author declares that he has no competing interests.

## Author’s contribution

 AJC is the single author of the paper.
